# Intermittent Hypoxia Affects the Spontaneous Differentiation *In Vitro* of Human Neutrophils into Long-Lived Giant Phagocytes

**DOI:** 10.1155/2016/9636937

**Published:** 2015-11-09

**Authors:** Larissa Dyugovskaya, Slava Berger, Andrey Polyakov, Peretz Lavie, Lena Lavie

**Affiliations:** The Lloyd Rigler Sleep Apnea Research Laboratory, Unit of Anatomy and Cell Biology, The Ruth and Bruce Rappaport Faculty of Medicine, Technion-Israel Institute of Technology, 31096 Haifa, Israel

## Abstract

Previously we identified, for the first time, a new small-size subset of neutrophil-derived giant phagocytes (G*ϕ*) which spontaneously develop *in vitro* without additional growth factors or cytokines. G*ϕ* are CD66b^+^/CD63^+^/MPO^+^/LC3B^+^ and are characterized by extended lifespan, large phagolysosomes, active phagocytosis, and reactive oxygen species (ROS) production, and autophagy largely controls their formation. Hypoxia, and particularly hypoxia/reoxygenation, is a prominent feature of many pathological processes. Herein we investigated G*ϕ* formation by applying various hypoxic conditions. Chronic intermittent hypoxia (IH) (29 cycles/day for 5 days) completely abolished G*ϕ* formation, while acute IH had dose-dependent effects. Exposure to 24 h (56 IH cycles) decreased their size, yield, phagocytic ability, autophagy, mitophagy, and gp91-*phox*/p22-*phox* expression, whereas under 24 h sustained hypoxia (SH) the size and expression of LC3B and gp91-*phox*/p22-*phox* resembled G*ϕ* formed in normoxia. Diphenyl iodide (DPI), a NADPH oxidase inhibitor, as well as the PI3K/Akt and autophagy inhibitor LY294002 abolished G*ϕ* formation at all oxygen conditions. However, the potent antioxidant, N-acetylcysteine (NAC) abrogated the effects of IH by inducing large CD66b^+^/LC3B^+^ G*ϕ* and increased both NADPH oxidase expression and phagocytosis. These findings suggest that NADPH oxidase, autophagy, and the PI3K/Akt pathway are involved in G*ϕ* development.

## 1. Introduction

Neutrophils, the first line of defense against invading pathogens and harmful particles, are known as professional phagocytes. Yet, increased neutrophil survival within tissues or in the circulation can promote persistent inflammation resulting in tissue injury and dysfunction [1]. During the last decade it has become increasingly evident that neutrophil activities go far beyond pathogen clearance while new unanticipated functions were recognized [2]. Moreover, concepts such as “neutrophil plasticity” and “neutrophil heterogeneity” have emerged [3], implying that there are conditions under which neutrophils may differentiate into discrete subsets with increased longevity and with new phenotypes and functions [2]. Thus, by exposing neutrophils to GM-CSF/IL-4/TNF-*α*, long-lived neutrophil subsets with efficient phagocytosis, increased production of reactive oxygen (ROS), IL-1, and IL-8 developed [4]. Also transforming or reprogramming into other cell types, like macrophages or dendritic cells (DCs), was demonstrated [5, 6]. Murine neutrophils cultured with GM-CSF, or upon recruitment to inflammatory or infectious sites, are differentiated into a hybrid population with prolonged life span, exhibiting dual features and functionality of neutrophils and DCs [7–9]. Notably, the existence of long-lived neutrophil subsets is suggested through their ability to modulate their survival response by both intrinsic host-derived and extrinsic factors, such as G-CSF, GM-CSF, IFN-*γ*, TNF-*α*, and/or pathogen-derived products, nucleic acids, or hypoxic environmental conditions [10]. Specifically, inflammatory sites tend to become depleted of oxygen. Thus, the concept of “inflammatory hypoxia,” in which inflammation and hypoxia are inseparably linked, was proposed [11, 12]. Both sustained and intermittent hypoxia (SH and IH) were shown to profoundly inhibit neutrophil apoptosis resulting in increased neutrophil survival and activation [13, 14]. Moreover, prolonged neutrophil survival was also evident in patients with obstructive sleep apnea (OSA), a morbidity associated with nightly IH resulting in intermittent blood hypoxemia resembling ischemia/reperfusion (I/R) and associated with increased ROS production, oxidative stress, and systemic inflammation [15, 16].

To adapt to hypoxia, cells undergo a metabolic shift by increasing the cellular dependency on anaerobic metabolism and activate autophagy for degrading damaged or unnecessary proteins and organelles. In neutrophils, autophagy plays a cell death role in inflammatory/infectious conditions and in tumors which are characterized by hypoxia. Of note, autophagy is vital in sensing oxidative stress and removing oxidatively damaged cellular components and is activated by stress or triggered by cytoplasmic overload of these proteins or organelles. It may also play a role in neutrophil differentiation [17] and has been shown to control the generation of neutrophils in the bone marrow [18]. Additionally, a specific mitochondrial autophagy—mitophagy—has been demonstrated as a selective mechanism to remove dysfunctional or damaged mitochondria [19]. One of the key proteins of autophagy activation and a prominent autophagosome marker is LC3B. Generation of autophagosome structures requires conversion of the cytosolic LC3BI to the lipidated LC3BII and its translocation to autophagosomes [20]. Autophagy is negatively regulated by mTOR while it requires phosphatidylinositol 3-kinase (PI3K) for inducing the autophagic machinery [20] and for generating NADPH oxidase-dependent ROS [21]. Autophagy is also enhanced in cardiac I/R injury but acts as a double-edged sword in I/R related pathological processes [22]. Hence, besides its detrimental effects, autophagy can protect cardiac myocytes against I/R injury [23]. However, the interplay between ROS, autophagy, and cell survival is complex, cell specific, and not entirely understood [24]. All in all, these findings suggest that ROS might be involved in autophagic processes in conditions associated with IH.

In a previous study we have shown, for the first time, that freshly isolated purified PMN from healthy subjects maintained in prolonged culture conditions without additional growth factors or cytokines give rise to a small subpopulation of G*ϕ* within 5–7 days [25]. These G*ϕ* are characterized by unique morphology, phenotype, and functions. They are vastly enlarged due to autophagocytosis of dead neutrophil remnants, are vacuolated, and contain phagolysosomes. They express a marker of specific neutrophil granules CD66b, a marker of azurophilic granules CD63, CD15, CD11b, and MPO, the gp91-*phox* subunit of NADPH, and autophagy markers (LC3B). Functionally, they actively take up particles as latex and opsonized zymosan and generate ROS in response to these particulate stimuli and to PMA. Interestingly, unlike fresh PMN, G*ϕ* which also intensively expressed CD68 scavenger receptor took up oxidized LDL (oxLDL) and generated ROS in response to stimulation with oxLDL. Additionally, specific autophagy inhibitors as 3-methyladenine (3-MA) or bafilomycin (BafA1) abolished G*ϕ* development, demonstrating the importance of autophagy to G*ϕ* development [25]. However, the exact factors which determine G*ϕ* formation remain to be unveiled.

To further elucidate the conditions and mechanisms involved in G*ϕ* formation, we sought to investigate the effects of various hypoxic treatments on G*ϕ* development. To gain further insights into G*ϕ* development in hypoxic conditions, PMN were also treated with various pharmacologic inhibitors for ROS generation and signalling pathways.

## 2. Materials and Methods

### 2.1. Isolation and Culture of Polymorphonuclear Cells (PMN) and the Development of Giant Phagocytes (G*ϕ*) in Culture

Blood samples were obtained from 28 (26 males/2 females) healthy nonsmoker volunteers with a mean age of 28.0 ± 6.6 years and BMI of 24.9 ± 3.9 Kg/m^2^. Sleep studies were performed on all subjects using the WatchPAT-200 device [26], to rule out occult sleep disordered breathing. All subjects had less than 5 oxygen desaturation (ODI) breathing events per hour which is considered a normal value. The protocol was approved by the local Human Rights Committee according to the Declaration of Helsinki, and all participants signed an informed consent form. Some of the subjects were tested from two to six times. PMN were isolated using two-step density gradients of equal volumes of Ficoll-Histopaque-1077 and Ficoll-Histopaque-1.119 (Sigma-Aldrich, Israel) according to manufacturer's instructions, followed by lysis of red blood cells with 0.2% sodium chloride for 30 sec on ice. PMN were cultured in RPMI-1640 medium supplemented with 10% heat inactivated FCS (Biological Industries, Beit HaEmek, Israel). Half of the growth medium was carefully replaced every 3 days. After 5–7 days, G*ϕ* were evident in culture. Depending on the donor, from each 10^6^ neutrophils plated, 100–200 G*ϕ* developed. As a control, in some experiments, the culture medium was supplemented with 30 ng/mL granulocyte macrophage-stimulating factor (GM-CSF) and 30 ng/mL IL-4 (R&D Systems, Minneapolis, MN). GM-CSF/IL-4 was added at each medium change. The LPS content in FCS was lower than 1.0 ng/mL and the addition of 1–10 ng/mL LPS to the culture medium did not affect G*ϕ* formation [25].

Of note, to validate the neutrophilic origin of G*ϕ*, in earlier experiments we also prepared a FACS-purified population of CD15/CD11b/CD63/CD66b neutrophils. Their development in culture was similar to that obtained from PMN isolated by Ficoll only. Moreover, by coculturing PMN with autologous monocytes, G*ϕ* did not develop. Thus, excluding the possibility that G*ϕ* arise from contaminating cells or cells other than mature PMN, we should also note that since the yield of G*ϕ* formed from neutrophils in culture is low (0.01–0.02% of cultured PMN in normoxia), biochemical and molecular measures are hard to implement [25]. Therefore in this study the analyses rely mostly on a follow-up by confocal microscopy.

### 2.2. *In Vitro* Intermittent (IH) and Sustained Hypoxia (SH) Protocol

Purified PMN (0.6 mL per well; 3 × 10^6^ cells/mL) were plated into 24-well plates after which they were exposed to normoxia, SH, or IH in custom-designed incubation chambers attached to an external O_2_-CO_2_-N_2_ computer-driven controller using BioSpherix-OxyCycler-C42 system (Redfield, NY, USA). This system enables creating periodic changes in external O_2_ concentrations that control air gas levels in each chamber individually as previously described [13]. Oxygen levels in the medium were determined by a fiber-optic dissolved oxygen electrode (BioSpherix, Redfield, NY, USA). The actual lowest % of O_2_ in the medium dropped to 5% during the hypoxic period for about 1.5 min, and this level of hypoxia was achieved after 15 min of incubation. In the reoxygenation period, O_2_ levels reached normoxic levels (20%) after 10 min of incubation. Carbon dioxide was held constant (5%) at all treatments. For modeling chronic IH, the purified PMN were exposed for 5 consecutive days to 29 IH cycles/day (approximately 12 h/day). Acute IH was induced by exposing PMN to 10 cycles (250 min), 29 cycles (12 h), or 56 cycles (approximately 24 h), each in the first day in culture. SH was employed for comparable times at 5% actual oxygen in the medium for the entire periods (250 min, 12 h, and 24 h). Thereafter, the hypoxia treated cells were transferred to normoxia for additional six days, after which various measures were performed. Control cells were maintained in normoxia for the entire period.

### 2.3. Confocal Laser Scanning Microscopy

Cytospins prepared from 7-day G*ϕ* were fixed with 4% paraformaldehyde and washed with PBS. For intracellular staining, cells were permeabilized with 0.5% Triton X-100 (Sigma-Aldrich, Israel) in PBS, at room temperature for 10 min. After blocking with 10% normal goat serum in RPMI-1640, cells were incubated overnight at 4°C using the following primary Abs (dilution 1 : 100) or the corresponding isotype controls: mouse monoclonal anti-CD66b Abs (80H3, AbD Serotec, Oxford, UK) and anti-cytochrome b-245 light chain (p22-*phox* identification, Clone 44.1, BioLegend, San Diego, CA), rabbit polyclonal anti-neutrophil elastase (NE) (Calbiochem, San Diego, CA), anti-LC3B Abs (Sigma, Israel), and anti-Nox2/gp91-*phox* Abs (ab131083, Abcam, UK). Isotype controls included purified mouse IgG1 (clone MG1-45) and IgG2 (clone MOPC-173, BioLegend, San Diego, CA) and rabbit IgG (Santa Cruz Biotechnologies, Santa Cruz, CA). Then, the cells were washed and incubated with 1/400 secondary antibodies CF 488A or CF 647 goat anti-rabbit IgG and/or CF 647 goat anti-mouse IgG (Biotium, Hayward, CA). After washing, slides were mounted with mounting medium containing 4′,6-diamidino-2-phenylindole (DAPI) for nuclear staining (Vectashield H-1000, Vector Lab. Inc., Burlingame, CA). Slides were analyzed by a confocal laser scanning system (LSM 700) using Nikon E600 (Japan) fluorescence microscope and Plan Apo X40 immersion oil objective. Cells' area and fluorescent intensities (FI) were integrated with Image J 1.49k Software (Wayne Rasband, NIH, USA). Data are presented as FI = Raw integrated density (sum of pixel values)/Area of cells.

### 2.4. Fluorescence Labeling of Cells

The fluorescence membrane stains PKH-26 (red) and PKH-67 (green) (Sigma-Aldrich) were used to label freshly isolated neutrophils according to manufacturer's instructions. The labeling vehicle provided by the kits (Diluent C) is an aqueous solution designed to maintain cell viability. Cells were washed in serum-free medium; the pellets were resuspended in 0.5 mL of PKH-26 or PKH-67 (1 : 500 in Diluent C) and incubated for 5 min at room temperature. Labeling was stopped by adding 0.5 mL FCS. Then, cells were washed three times with complete medium and cocultured.

### 2.5. Phagocytosis

The phagocytic activity of G*ϕ* was determined on day 7 using fluorescent latex beads of 1.0 *μ*m in diameter (phagocytosis is considered when the particles internalized are about 0.75 *μ*m or larger). Briefly, G*ϕ* were incubated for 2 h with carboxylate-modified fluorescent yellow-green latex beads (Polyscience, Warrington, PA) at a cell : bead ratio of 1 : 500 (because of the large G*ϕ* cell size). Cytospins were prepared and fixed as described above and analyzed by confocal microscopy. Three types of controls were performed to ensure intracellular localization of the beads. (1) Control G*ϕ* were kept on ice for 15 min and cytospins were prepared immediately or 2 h after adding the latex beads. (2) To inhibit phagocytosis, G*ϕ* were preincubated with 10 *μ*M cytochalasin B (Sigma-Aldrich, Israel) for 30 min prior to adding the latex beads, and cytospins were prepared 2 h after incubation with latex. (3) To ensure intracellular localization rather than adhesion, the intracellular localization of latex beads was confirmed in G*ϕ* by 3D images (*xy*, *xz*, and *yz* cross sections) using 3D reconstructing software IMARIS* z*-stack analysis (Bitplane AG, Switzerland), and only latex beads in the plane of the nucleus were considered positive for phagocytosis.

### 2.6. Lysosomal and Mitochondrial Distribution

LysoTracker (Invitrogen, Molecular Probes, Eugene, Oregon, USA) was used to detect acidified endosomes. Viable G*ϕ* were incubated with 50 nM of LysoTracker for 90 min at 37°C in the dark. To identify mitochondria, viable cells were stained for 30 min at 37°C in the dark with 100 nM MitoTracker Orange CMTMRos (Invitrogen, Molecular Probes, Eugene, Oregon, USA). To further detect mitophagy, fixed cytospins were stained with LC3B, as described above.

Colocalization was quantified by ZEN 2010 (version 6.0) Carl Zeiss MicroImaging GmbH, Germany using Manders Overlap Coefficient (MOC) [27]. Only cells with MOC > 0.6 were considered as cells with significant colocalization.

### 2.7. Treatments of Neutrophils with Inhibitors

Freshly isolated PMN were exposed for 24 h to IH, SH, or normoxia with or without various inhibitors. Each inhibitor was added 10 min prior to the various oxygen treatments and remained throughout the treatments. The following inhibitors were used: a NADPH oxidase inhibitor, 10 *μ*M diphenyl iodide (DPI); a ROS scavenger, 20 *μ*M N-acetylcysteine (NAC) (all purchased from Sigma-Aldrich, St. Louis, MO, USA); and PI3K inhibitor LY-294002, 20 *μ*M (L-1023, A.G. Scientific, San Diego, CA, USA) which is indicated to act at this concentration as a PI3K inhibitor not affecting TLR signaling cascade. Equal volumes of DMSO were used as a negative control.

### 2.8. NBT Test

Intracellular ROS was determined in 7-day G*ϕ* by NBT test. NBT (Sigma-Aldrich) was dissolved in RPMI 1640 without phenol red (0.2%). Cells were incubated without or with 100 nM PMA at 37°C for 15 min, then kept at room temperature for 10 min, and assessed by light microscopy. In some experiments also DPI was added to G*ϕ*, 2 h prior to PMA stimulation. Cytoplasmic clumps of formazan deposits in G*ϕ* were considered positive for ROS.

### 2.9. Cell Viability by WST-1 Assay

Cell viability was monitored in the various oxygen and inhibitor treatments by using a commercial reagent WST-1 (Roche Diagnostics GmbH, Mannheim, Germany), according to manufacturer's instructions [28]. Briefly, purified PMN were exposed for 24 h duration to normoxia, SH or IH with or without various inhibitors (see above). Then cells were seeded at a concentration of 5 × 10^4^ cells/well in 100 *μ*L culture medium (tissue culture grade, 96 wells, flat bottom) and were incubated with the WST-1 reagents (10 *μ*L/well) for 1 h. The formazan dye formed was determined with ELISA reader at 450/650 nM. The measured absorbance directly correlates to the number of viable cells.

### 2.10. SDS-PAGE and Western Blot Analysis

Cells were washed twice and extracts were prepared in lysis buffer containing 50 mM Tris-HCl (pH 7.4), 150 mM NaCl, and 1% NP-40 supplemented with a mixture of protease inhibitors (Roche Applied Science). Protein concentration was determined using the Bradford reagent (Bio-Rad), and 30 *μ*g of protein was loaded onto SDS-PAGE under reducing conditions. After electrophoresis, proteins were transferred to polyvinylidene difluoride (PVDF) membrane (Bio-Rad) and probed with rabbit polyclonal antibody to LC3B (Sigma, Saint Louis, USA), followed by horseradish peroxidase-conjugated secondary antibody (Jackson ImmunoResearch Laboratories, Inc., West Grove, PA) and an enhanced chemiluminescent substrate (Beit HaEmek, Israel). Densitometric analysis was performed using TotalLab TL100 v.2006c software (Nonlinear Dynamics Ltd., Newcastle Upon Tyne, UK). Data were normalized over *β*-actin and the ratio of LC3BII/LC3BI was also calculated.

### 2.11. Statistical Analysis

Data are expressed as mean ± SD for each experimental group. A two-tailed Student's* t*-test with Bonferroni correction was used for multiple comparisons. Therefore, only values of *p* < 0.008 were considered significant. The NCSS 2004 statistical package (Kaysville, Utah) was used.

## 3. Results and Discussion

### 3.1. Development of G*ϕ* in Normoxia

**Figure 1:**
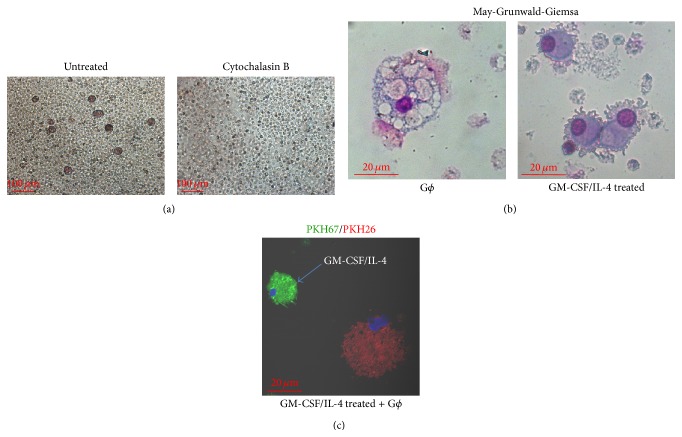
Effects of cytochalasin B and GM-CSF/IL-4 on giant phagocyte (G*ϕ*) formation. (a) Freshly isolated PMN were incubated at time 0 without or with 10 *μ*M cytochalasin B and followed for 7 days in culture. Bright-field microscopy of 7-day living cultures. (b) Representative photomicrographs of May-Grünwald-Giemsa-stained cytospin preparations of PMN cultured without (G*ϕ*) or with GM-CSF/IL-4 for 7 days. Samples were analyzed with a bright-field microscopy. Magnification, ×40. Cells developed in cultures with medium supplemented with GM-CSF/IL-4 show widespread cytoplasmic projections. Representative data out of 3 independent experiments. (c) PKH-26 (red) dye labeled PMN were cultured in cytokine-free medium for 7 days and PKH-67 (green) dye labeled PMN were cultured in medium supplemented with GM-CSF/IL-4 for 7 days. Then the developed cells were mixed in a ratio of 1 : 1 and cocultured for 2 h. Cytospins were fixed and analyzed by confocal microscopy.

Previously we have shown that, under normoxic conditions, a new neutrophil-derived subpopulation of cells characterized by neutrophilic markers CD15/CD11b/CD63/CD66b, as well as a unique morphology and functions, spontaneously develop into G*ϕ* under prolonged culture conditions [25]. Accordingly, in this study, G*ϕ* which developed in normoxic conditions had the same characteristics of G*ϕ* as previously shown (CD66b^+^, LC3B^+^,* gp91-phox* expression, large phagolysosomes, and phagocytosis). These G*ϕ* avidly phagocytosed neutrophil remnants, including granules and microparticles, suggesting that this active phagocytosis of neutrophil remnants may induce activation of some neutrophil subsets or precursors resulting in their transformation into G*ϕ* [25]. Thus, we treated PMN with cytochalasin B, which inhibits phagocytosis by inhibiting the polymerization of actin and prevents phagosome closure. As illustrated in Figure 1(a), cytochalasin B abolished the formation of G*ϕ*, indicating the importance of autophagocytosis to their development.

In addition, for comparison, we also followed PMN cultures supplemented with GM-CSF/IL-4. These cells were shown to develop into various cell types after 7–14 days in culture as previously described [9, 29]. Unlike G*ϕ*, the cells which developed in GM-CSF/IL-4 supplemented medium were mostly smaller in size, showed widespread cytoplasmic projections (Figure 1(b)), and were negative or had a low CD66b expression (data not shown). Morphologically, they resembled DC-like cells as reported by Oehler et al. [29] or the murine neutrophil-DC “hybrid” population demonstrated by Matsushima et al. [9].  Figure 1(c) illustrates the size differences between G*ϕ* and the cells which developed in GM-CSF/IL-4 supplemented medium. We divided freshly isolated PMN into two, and half of PMN were labeled with PKH-26 (red) dye and cultured in cytokine-free medium for 7 days, while the other half of PMN were labeled with PKH-67 (green) dye and cultured in GM-CSF/IL-4 supplemented medium for 7 days. Then, both cell types were mixed in a 1 : 1 ratio and cocultured for 2 h and cytospins were prepared. Notably, G*ϕ* formation is dependent on the local cytokine milieu, and culturing neutrophils with GM-CSF/IL-4 did not induce G*ϕ* formation but rather a different long-lived subpopulation. Since cells obtained in GM-CSF/IL-4 supplemented cultures did not resemble G*ϕ*, we did not follow their phenotypic and functional characteristics.

### 3.2. Development of G*ϕ* under Hypoxic Conditions

**Figure 2:**
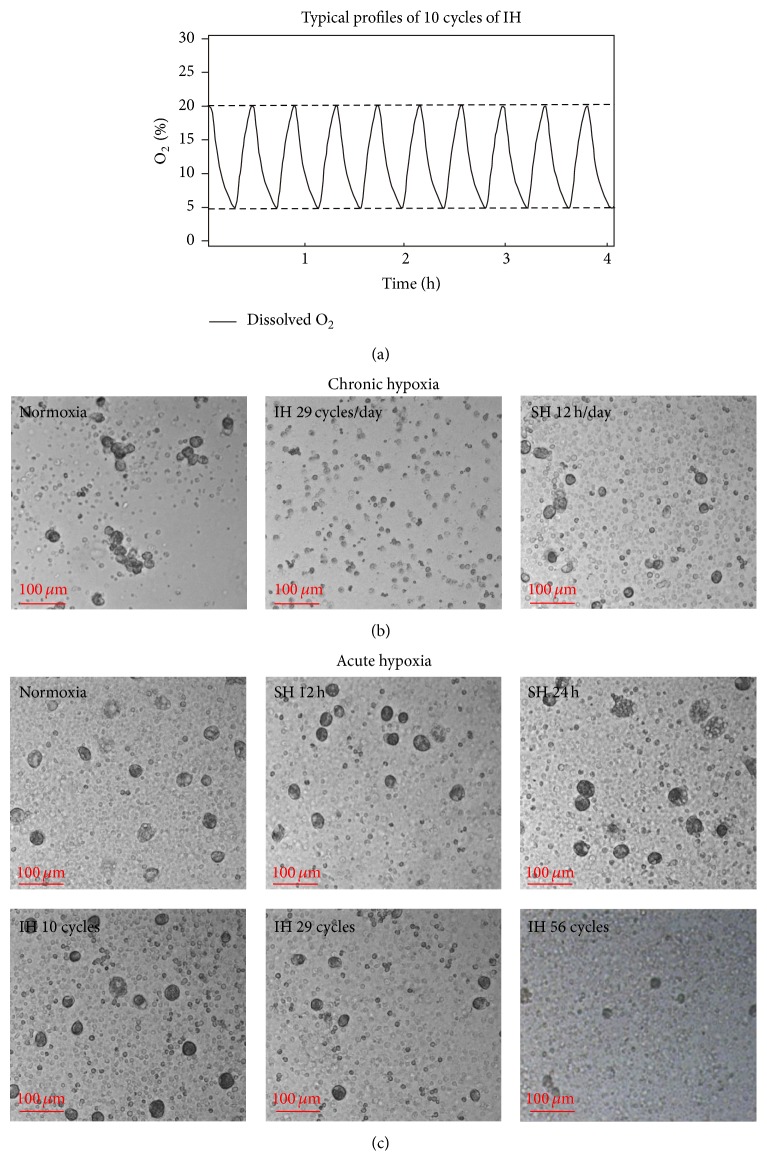
Effects of chronic and acute hypoxia on the development of giant phagocytes (G*ϕ*). (a) A typical profile of 10 intermittent hypoxia (IH) cycles. During IH actual % O_2_ in the medium (solid line) decreased to 5% oxygen concentration during the hypoxia. In the reoxygenation period O_2_ levels reached 20% oxygen. (b) For chronic hypoxia treatments, PMN were exposed to 29 cycles (approximately 12 h) of IH/day for 5 days or to a comparable time of sustained hypoxia (SH)/day. Controls were maintained at normoxia for the entire period. (c) For acute hypoxia treatments, PMN were exposed to 10 IH cycles (250 min), 29 IH cycles (approximately 12 h), or 56 IH cycles (approximately 24 h), each in the first day in culture. SH was employed for comparable times at 5% actual oxygen in the medium for the entire period (12 h and 24 h) and control cells were maintained at normoxia. Thereafter, the hypoxia treated cells were transferred to normoxia for additional six days. Light microscopy of living culture. Representative data out of 3 independent experiments. In (b) and (c) is bright-field microscopy of living cells.

Hypoxic environments are common in inflammatory and other pathological conditions. Neutrophils adapt to such hypoxic-pathological environments for the resolution of inflammation by relying on their unique molecular features such as preferential glycolysis as an ATP source over the mitochondria and a potent NADPH oxidase-dependent machinery producing massive amounts of ROS [30]. Thus, freshly isolated purified PMN from healthy subjects were exposed to various acute and chronic hypoxic conditions and compared to normoxia, as indicated in Materials and Methods. A typical profile of 10 IH cycles is presented in Figure 2(a). In the chronic IH protocol PMN were exposed to 29 cycles (12 h) of IH/day for five consecutive days. PMN were also maintained in SH (12 h/day for 5 days) and normoxia for the same period of time. As shown in Figure 2(b), treatment with chronic IH completely abolished G*ϕ* formation, but their development was evident in SH and in normoxic cultures. Thus, in the following experiments we focused on acute IH treatments for G*ϕ* development.

In the acute IH protocol, purified PMN were exposed to 10 cycles (250 min), 29 cycles (approximately 12 h), or 56 cycles (approximately 24 h) of IH, each on the first day in culture. In parallel cells were also exposed to corresponding times of SH (250 min, 12 h, and 24 h). Subsequently, PMN were cultured during the following 6 days at normoxic conditions. Control cells were maintained in normoxia for the entire durations.

Exposing PMN to 10 cycles of IH (250 min) had no effect on G*ϕ* development (Figure 2(c)). However, exposing PMN to 29 cycles of IH slightly decreased the quantity and size of the developed G*ϕ*. By exposure to 56 cycles of IH, G*ϕ* size was further decreased by about 30% (*p* < 0.005 (Table 1)) and the yield was only 20–25% of that obtained in controls. In SH, cell yield and size were unaffected (Table 1), although some variation in G*ϕ* cell size was noted after 24 h of SH. Confocal microscopy revealed that most of the SH-treated G*ϕ* had a normoxic appearance but some were smaller or slightly bigger than the control cells (Figures 2(b) and 2(c)). Moreover, two of the subjects were studied twice one month apart with similar results in both experiments. Jointly, these findings raise the possibility that, at sites of “inflammatory hypoxia,” neutrophils may adapt to SH and transform into G*ϕ* able of removing dead cells and/or cell debris, possibly when the macrophage/DCs system is insufficient. The autophagocytosis of apoptotic PMN remnants by the developing G*ϕ*, which we have previously demonstrated, is in accord with this possibility [25]. However, in IH which is a signature feature of I/R injury in a great number of pathologies and induces activation of various leukocytes [13, 15, 16], exposure of PMN to prolonged acute IH in the first day in culture might have switched the cells' program towards inhibiting G*ϕ* formation.

**Figure 3:**
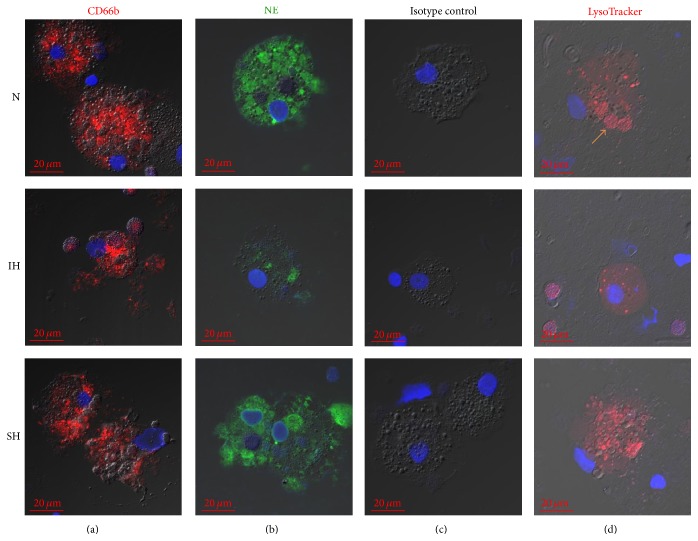
Effects of acute hypoxia on the development of giant phagocytes (G*ϕ*). Freshly isolated PMN were exposed for 24 h to intermittent hypoxia (IH, 56 cycles), sustained hypoxia (SH), or normoxia (N) and then cultured at normoxia for additional six days. Cytospins were prepared and analyzed by confocal microscopy (see Materials and Methods). Nuclei were stained with DAPI (blue). (a) Fixed cytospins were stained with anti-CD66b Abs and (b) neutrophil elastase (NE). (c) Isotype controls: fixed cytospins were stained with mouse IgG2 followed by 1/400 CF 647 goat anti-mouse IgG (red) staining. (d) Viable cells were stained with LysoTracker before fixation (see Materials and Methods). Arrow indicates large phagolysosomes. Representative data out of 3 independent experiments.

Since acute exposure to 56 IH cycles (24 h IH) had the strongest effect on G*ϕ* formation, it was chosen to further investigate G*ϕ* phenotype and functions. As shown in Figure 3(a), G*ϕ* formed at all oxygen treatments were CD66b positive. The developed G*ϕ* were also neutrophil elastase (NE) positive, indicating that G*ϕ* express this important neutrophil specific protein after differentiation. However, the expression of NE was significantly lower in IH-treated G*ϕ* compared to G*ϕ* formed in normoxia or SH. Although in SH-treated G*ϕ* NE levels were also attenuated, they did not significantly differ from controls (Table 1, Figure 3(b)). By staining G*ϕ* with LysoTracker (Figure 3(d)), a stain commonly used as a lysosomal marker exhibiting a proportional fluorescence to the volume of lysosome-related organelles in a cell [31], a different lysosomal morphology was noted between the various acute oxygen treatments. Unlike the large phagolysosomes formed in normoxia- or SH-treated G*ϕ*, the phagolysosomes of IH-treated G*ϕ* were small, and only small spots of lysosome-like structures were stained by LysoTracker. Also the intensity of LysoTracker staining was significantly lower by nearly 70% in these IH-treated G*ϕ* (Table 1), indicating lysosomal dysfunction.

All in all, G*ϕ* development under SH treatments basically resembled the development of normoxia-treated G*ϕ*, mainly with regard to size, CD66b, and LysoTracker expression. Yet other measures as NE varied. However, under IH, G*ϕ* development was significantly attenuated in a severity-dependent manner.

### 3.3. Effects of Hypoxia on Autophagy

**Figure 4:**
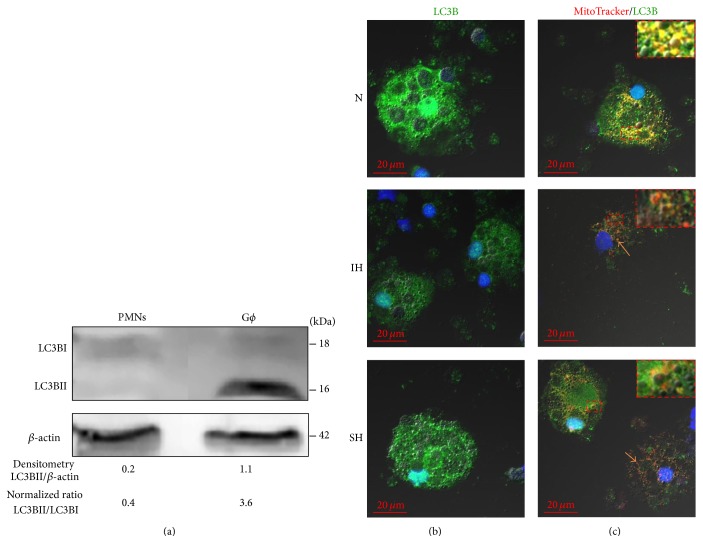
Effects of acute hypoxia on LC3B expression in giant phagocytes (G*ϕ*). Freshly isolated PMN were exposed for 24 h to intermittent hypoxia (IH, 56 cycles), sustained hypoxia (SH), or normoxia (N) and then cultured at normoxia for additional six days. (a) A representative western blot for LC3BI and LC3BII expression in freshly isolated PMN and normoxic-G*ϕ*. Densitometric analysis of LC3BII normalized over *β*-actin and normalizing by LC3BII/LC3BI ratio are presented. (b) Fixed cytospins were stained with LC3B (see Materials and Methods). Nuclei were stained with DAPI (blue). (c) Viable cells were labeled with MitoTracker Orange CMTMRos following fixation and LC3B staining. Mitophagy (LC3B positive structures containing mitochondria) appears as yellow spots (inserts in N and SH) with significant colocalization (MOC > 0.6) of LC3B/MitoTracker. Mitochondria without LC3B (red dots, arrows) were mainly noted in IH-G*ϕ* but also in SH-G*ϕ*. Representative data out of 4 independent experiments.

Autophagy represents an adaptive response to various stresses such as starvation, hypoxia, and excessive ROS by regulating cell death/survival, phagocytosis of dead cells, and contributing to neutrophil differentiation [17]. We have previously shown that, under normoxia, autophagy is a constitutive trait of G*ϕ* by demonstrating the presence of LC3B-II as vesicular puncta associated with autophagosomes and that treatment with specific autophagy inhibitors abolished G*ϕ* formation [25]. Herein we further confirmed the expression LC3B-II in G*ϕ* at the protein level as illustrated in Figure 4(a). Densitometric analysis of LC3BII normalized over *β*-actin and normalizing by LC3BII/LC3BI ratio revealed a high LC3BII expression in G*ϕ* compared to fresh PMN, clearly indicating active autophagy in G*ϕ*. Examples of G*ϕ* stained for LC3B at the various oxygen treatments are depicted in Figure 4(b). Unlike the case in normoxia, LC3B expression was significantly lowered by about 40% in 24 h IH-treated G*ϕ* (Table 1). Additionally, also in SH-treated G*ϕ* LC3B levels were attenuated; however, they did not significantly differ from controls (*p* = 0.1) (Table 1). These findings clearly suggest that autophagy might be decreased in G*ϕ* to various degrees depending on the type of the hypoxia inflicted.

Mitochondrial autophagy (mitophagy) mediates the selective elimination of dysfunctional or unwanted mitochondria [32]. Therefore, the effects of 24 h IH on mitophagy were investigated by double labeling for the autophagosomal compartment LC3B (green) and the mitochondria with MitoTracker (red). Two types of G*ϕ* were noted (Figure 4(c)). In normoxia, G*ϕ* expressing LC3B positive structures containing mitochondria (mitophagy) were the predominant G*ϕ* type (57.4 ± 7.2% expressed mitophagy, *n* = 4). These appeared as yellow spots with significant colocalization of LC3B and MitoTracker (MOC > 0.6). In SH, 34.8 ± 2.8% expressed mitophagy, but the morphology was mixed (Figure 4(c), inserts in normoxia and SH). However, in the 24 h IH-treated cells, the predominant type had mitochondria without LC3B (only 3–6% of G*ϕ* expressed mitophagy). Thus, in IH, mitochondria and LC3B were mostly not colocalized (Figure 4(c) red dots, arrow). Also, the intensity of MitoTracker expression in 24 h IH-treated G*ϕ* was 59.7 ± 25.6% of that expressed in normoxic-G*ϕ*, indicating the loss of membrane potential (Table 1). These findings indicate that, in the IH-treated G*ϕ*, mitochondria are dysfunctional and display an abnormal mitophagy.

### 3.4. Effects of Hypoxia on the Cellular Localization of gp91-*phox* and p22-*phox* and Its Involvement in G*ϕ* Development

**Figure 5:**
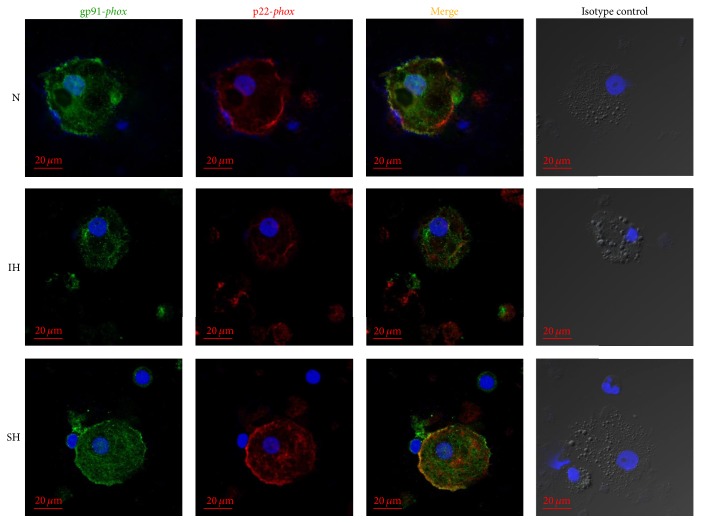
Effects of hypoxia on gp91-*phox* and p22-*phox* expression in giant phagocytes (G*ϕ*). Freshly isolated PMN were exposed for 24 h to intermittent hypoxia (IH, 56 cycles), sustained hypoxia (SH), or normoxia (N) and then cultured at normoxia for additional six days. For double immunofluorescence staining fixed cytospins were stained with rabbit anti-gp91-*phox* and mouse anti-p22-*phox* primary Abs (1/100) or the corresponding isotype controls (rabbit IgG and mouse IgG2) followed by 1/400 CF 488A goat anti-rabbit IgG (green) and CF 647 goat anti-mouse IgG (red) staining. Nuclei were stained with DAPI (blue). Representative data out of 3 independent experiments.

NADPH oxidase-derived ROS are a key signal for autophagy through LC3 recruitment to phagosomes [33]. In resting inflammatory cells NADPH oxidase is predominantly inactive and its components are separately distributed between the cytosol and the membranes. However, upon stimulation, its subunits (gp91-*phox*, p22-*phox*, p-47-*phox*, p67-*phox*, p40-*phox*, and Rac2) are assembled as the functional NADPH oxidase at the phagosomes and/or the plasma membrane [34–37]. We therefore determined the expression of two of its critical subunits, namely, gp91-*phox* and p22-*phox.* The intracellular localization of gp91-*phox* (green) and p22-*phox* (red) was determined by double immunofluorescence staining, as depicted in Figure 5. The gp91-*phox* subunit was highly expressed in normoxic-G*ϕ* and as expected was localized in the plasma and phagolysosome membranes as previously shown [25]. In 24 h SH-treated G*ϕ* the gp91-*phox* expression was on average lower than that of normoxia. However, in 24 h IH-treated G*ϕ*, the expression of gp91-*phox* was significantly lowered to 28.4 ± 15.4 of normoxic-G*ϕ* (*n* = 5). Typical examples of gp91-*phox* fluorescence intensity of expression at different oxygen conditions are depicted in Table 1.

**Figure 6:**
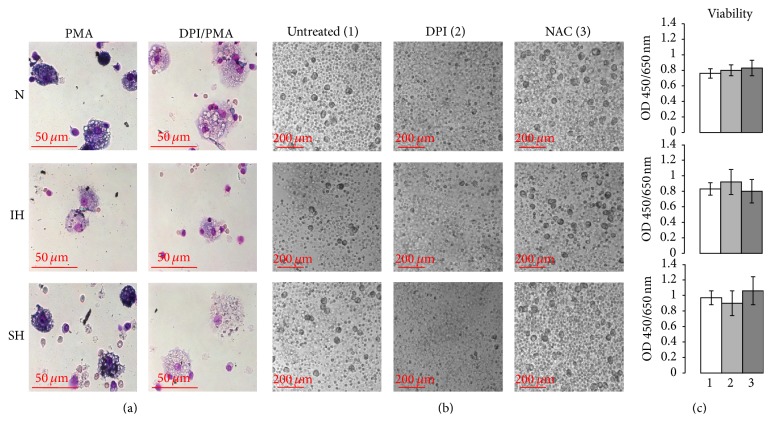
PMA-dependent ROS production, and the effects of diphenyl iodide (DPI) (10 *μ*M) and N-acetylcysteine (NAC) (20 *μ*M) on viability and development of giant phagocytes (G*ϕ*). PMN were cultured during 24 h in normoxia (N), intermittent hypoxia (IH) (56 cycles), or sustained hypoxia (SH) without or with inhibitors and then cultured at normoxia for additional six days. (a) Intracellular ROS production was detected in PMA-activated G*ϕ* by NBT test (see Materials and Methods). DPI was added to G*ϕ* 2 h prior to PMA stimulation. Bright-field microscopy of Giemsa-stained cultures in the various oxygen treatments. (b) DPI or NAC were added to PMN cultures 10 min prior to exposing to N, IH, and SH. Then PMN were cultured during the next 6 days at normoxia. Equal volumes of DMSO were added as a negative control. Bright-field microscopy of living cultures in the various treatments. (c) PMN viability was detected immediately after 24 h of the hypoxic treatments in untreated (1), DPI treated (2), or NAC-treated cells (3) using WST-1 assay. The measured absorbance (OD) directly correlates to the number of viable cells in each treatment.

The p22-*phox* subunit was also mainly localized in the plasma membranes and phagolysosomes in normoxic and in 24 h SH-treated G*ϕ*. However, while, in 24 h IH-treated G*ϕ*, the intensity of the p22-*phox* subunit expression was lowered to 45% of normoxic-G*ϕ*, in 24 h SH-treated G*ϕ* its intensity of expression was significantly increased to 165% compared to normoxic-G*ϕ* (*p* < 0.01). Typical examples of p22-*phox* fluorescence intensity of expression at different oxygen conditions are depicted in Table 1. Colocalization of gp91-*phox* with p22-*phox* was evident in the cell membranes of normoxic- and 24 h SH-treated G*ϕ* (Figure 5). However, in 24 h IH-treated G*ϕ* these subunits were rarely colocalized but rather were located separately. The inability of NADPH oxidase to undergo assembly in response to IH may indicate that the enzyme is not activated in IH-treated G*ϕ* and thus its signaling properties might be altered. This is clearly evidenced by the lower NBT dye reduced into formazan in IH-treated G*ϕ* that were stimulated by PMA to produce ROS, compared to normoxia- and SH-treated G*ϕ* (Figure 6(a)). Moreover, inhibiting NADPH oxidase with DPI, prior to PMA stimulation, confirms the data regarding NADPH oxidase distribution and colocalization in the various oxygen treatments (in Figure 8(b)) but also indicates that NADPH oxidase is a ROS contributor in normoxia and SH and less so in IH, thus, likely maintaining its signaling properties in normoxia and SH, but not in IH.

To explore the potential involvement of NADPH oxidase in G*ϕ* development, 10 *μ*M DPI was added to fresh PMN cultures 10 min prior to the exposure to the various 24 h oxygen treatments. After 7 d in culture the development of G*ϕ* was completely abolished at all oxygen conditions (Figure 6(b)), but it did not affect cell viability after 24 h in culture as determined with WST-1 test (Figure 6(c)). Although DPI is an inhibitor of flavin containing enzymes and mitochondria, it was shown to be less potent for mitochondrial oxidative phosphorylation and other flavin enzymes than for NADPH oxidase. Moreover, mitochondria are scarce in neutrophils. Therefore the effects of DPI are mostly attributed to inhibition of NADPH oxidase [38].

Jointly, the lower expression and assembly of NADPH oxidase as well as its lower production of ROS in IH-treated G*ϕ* and its inhibition by DPI at all oxygen conditions which prevented G*ϕ* formation may suggest that NADPH oxidase-dependent ROS production contributes to G*ϕ* formation, likely through signaling pathways that regulate autophagy. Possibly, decreased NADPH oxidase expression in IH-treated cells might prevent LC3B recruitment or alter NADPH-dependent ROS signaling pathways such as PI3K [39] and therefore mitigate the autophagy-depend G*ϕ* development.

### 3.5. Effects of PI3K Inhibitor on G*ϕ* Development

The PI3K specific inhibitor LY-294002 was added at 20 *μ*M to PMN cultures 10 min prior to the exposure to the various 24 h oxygen treatments (this concentration does not inhibit the TLR signaling cascade). Equal volumes of DMSO were added as a negative control. Inhibiting the PI3K kinase pathway with LY-294002 abolished G*ϕ* formation at all oxygen conditions studied (data not shown). Also, inhibition of class III PI3K by 3-methyladenine (3-MA), a commonly used autophagy inhibitor, was previously shown to inhibit G*ϕ* formation [25]. Jointly, these findings support the involvement of PI3K pathway in the formation of G*ϕ*.

The PI3K/Akt signaling pathway is essential for several neutrophil functions, including migration, degranulation, and superoxide production by controlling NADPH oxidase activation [40–42]. Akt phosphorylates p47*phox*, facilitating its membrane translocation and activation [41]. Akt is also a well-established inhibitor of apoptosis and inhibiting Akt promotes apoptosis [43]. Thus, it is likely that PI3K/Akt may act as an indispensable pathway for G*ϕ* formation via controlling NADPH oxidase activation.

### 3.6. Effects of NAC on G*ϕ* Development

**Figure 7:**
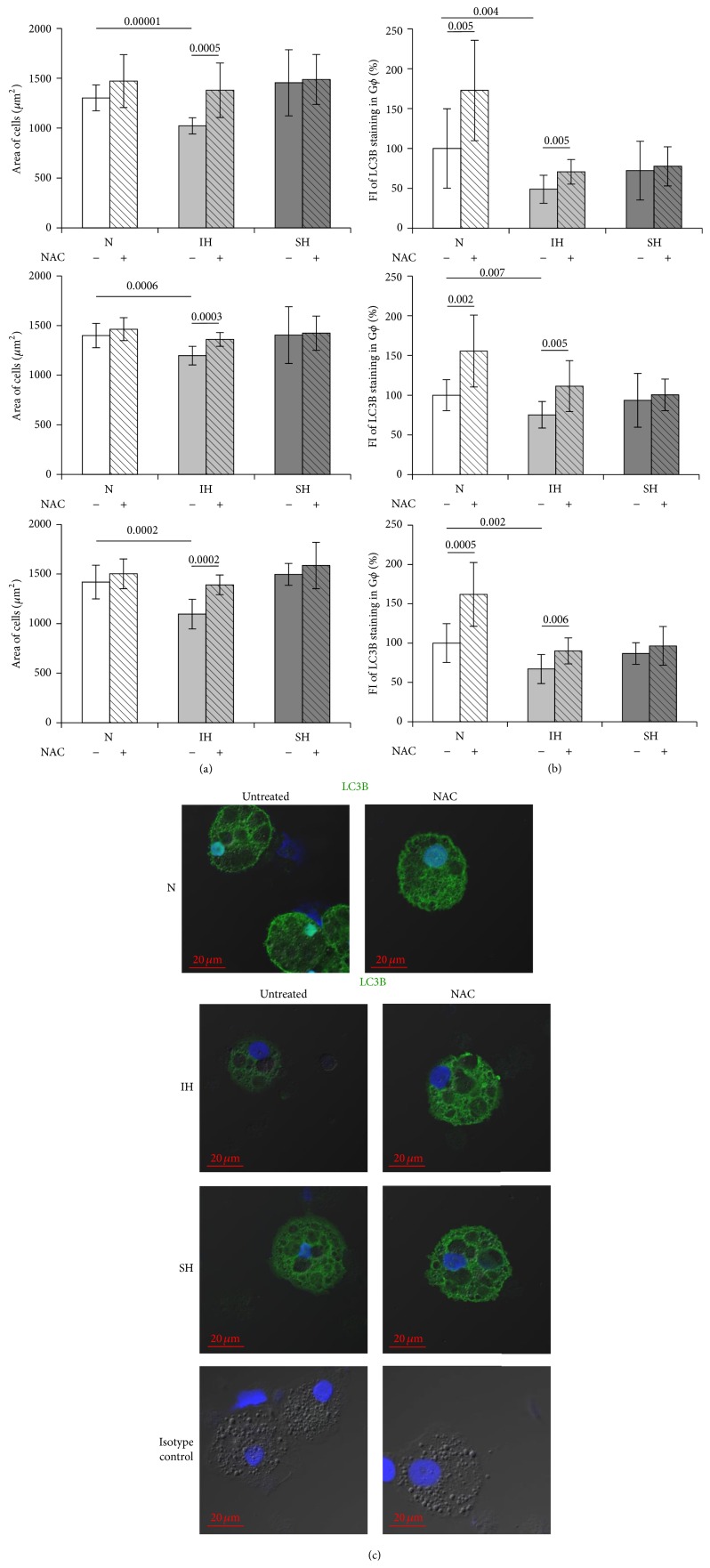
Effects of N-acetylcysteine (NAC) on giant phagocytes (G*ϕ*) size and LC3B expression. Freshly isolated PMN were exposed for 24 h to intermittent hypoxia (IH, 56 cycles), sustained hypoxia (SH), or normoxia (N) and then cultured at normoxia for additional six days. NAC (20 *μ*M) was added to PMN cultures 10 min prior to exposing to N, IH, or SH. Equal volumes of DMSO were added as a negative control. Fixed cytospins were stained with rabbit anti-LC3B primary Abs (diluted 1/100) or corresponding isotype controls (rabbit IgG) followed by 1/400 CF 488A anti-rabbit IgG staining (green). Nuclei were stained with DAPI (blue). (a) Area of cells (*μ*m^2^) in three independent experiments. (b) Fluorescence intensity (FI) of LC3B expression, integrated with Image J Software (see Materials and Methods), in three independent experiments. (c) Representative photomicrographs of G*ϕ* which developed after exposure to N, IH, or SH without (untreated) or with NAC. Isotype controls: untreated or NAC-treated normoxic-G*ϕ*.

**Figure 8:**
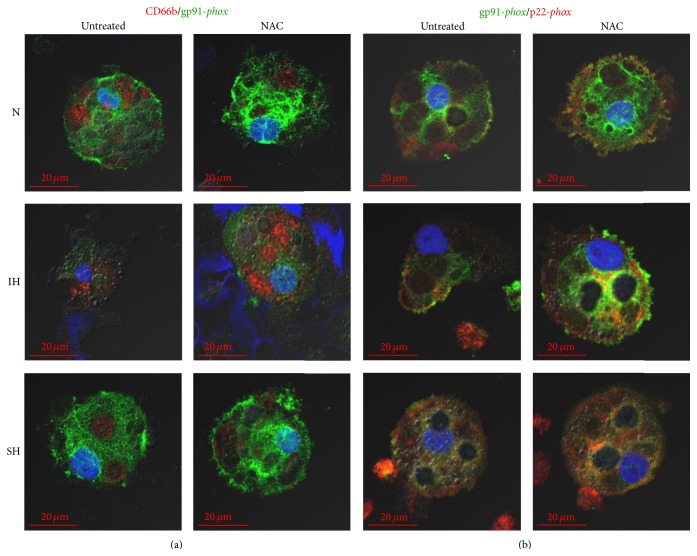
Expression of NADPH oxidase subunits in giant phagocytes (G*ϕ*) and the effects of NAC on their expression. Freshly isolated PMN were exposed for 24 h to intermittent hypoxia (IH, 56 cycles), sustained hypoxia (SH), or normoxia (N) and then cultured at normoxia for additional six days. NAC (20 *μ*M) was added to PMN cultures 10 min prior to exposing to N, IH, or SH. Equal volumes of DMSO were added as a negative control. Cytospins were prepared and analyzed by confocal microscopy (see Materials and Methods). The developed G*ϕ* were stained by double immunofluorescence: (a) for CD66b (red) and gp91-*phox* (green) in untreated and NAC-treated G*ϕ* and (b) for gp91-*phox* (green) and p22-*phox* (red) in untreated and NAC-treated G*ϕ*. Nuclei were stained with DAPI. Representative photomicrographs out of 3 independent experiments.

To further probe the potential involvement of ROS in G*ϕ* development, the potent antioxidant and glutathione precursor—NAC—was used [44]. NAC (20 *μ*M) was added to PMN cultures 10 min prior to the exposure to 24 h of normoxia, SH, or IH. Viability of the cells in the presence of NAC, measured after 24 h, was unaffected at all three oxygen conditions (Figure 6(c)). Treatment with NAC did not affect G*ϕ* development in normoxia or in SH. However, it had a robust impact on IH-treated PMN cultures exposed to 56 cycles. After 7 days in culture morphology and functions were altered; the G*ϕ* size and LC3B expression were significantly increased and resembled normoxia-treated cells (Figures 7(a)–7(c)). Treatment with NAC also increased CD66b expression and phagolysosome size (Figure 8(a)). Additionally, the expression of gp91-*phox* and p22-*phox* subunits and their redistribution in cell membranes, as well as around phagosomes in these 24 h IH-treated G*ϕ*, resembled those maintained in normoxia (Figure 8(b)). Thus, treatment with NAC abolished the effects of IH and partially restored the normoxic phenotype. Treatment with NAC twice weekly was also shown to promote hematopoietic differentiation of induced pluripotent stem cells (iPSCs) in long-term culture by mitigating oxidative stress [45]. Accordingly, in our study, treatment with NAC increased G*ϕ* size and punctuation of LC3B in the IH-treated G*ϕ* already after 2 days in culture (data not shown). It is thus suggested that the presence of NAC can restore the cellular ROS balance by replenishing the intracellular glutathione levels during IH and therefore facilitate the development of G*ϕ* with similar characteristics to those obtained in normoxia with regard to size, LC3B, and NADPH oxidase expression.

### 3.7. Effects of Hypoxia and NAC on the Phagocytic Activity of G*ϕ*

**Figure 9:**
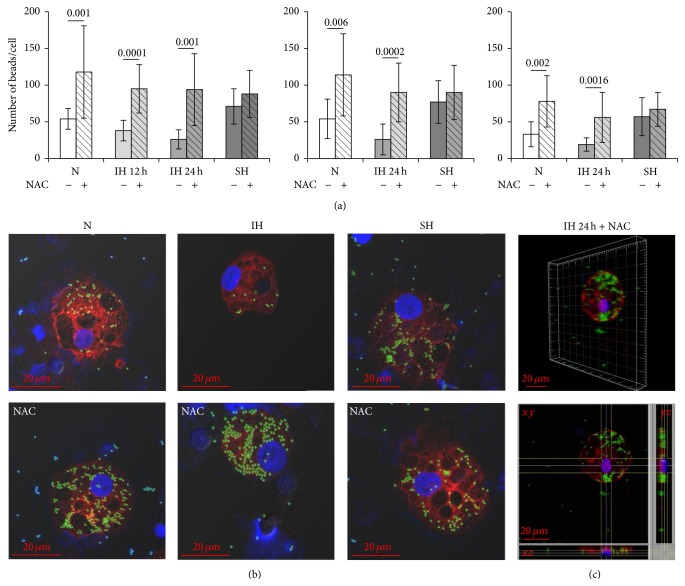
Phagocytic activity of giant phagocytes (G*ϕ*) and the effects of NAC on G*ϕ* phagocytosis. Freshly isolated PMN were exposed for 12 h (29 cycles) or 24 h (56 cycles) to intermittent hypoxia (IH), 24 h sustained hypoxia (SH), or normoxia (N) and then cultured at normoxia for additional six days. NAC (20 *μ*M) was added to PMN cultures 10 min prior to exposing to N, IH, or SH. The developed G*ϕ* (7 d culture) were incubated for 2 h with carboxylate-modified fluorescent yellow-green latex beads. Cytospins were prepared, fixed, stained with LC3B (red), and analyzed by confocal microscopy (see Materials and Methods). Nuclei were stained with DAPI. (a) The average number of beads/cell in G*ϕ* developed after exposure to N, IH, or SH without or with NAC in three independent experiments. In each experiment at least 10 cells were counted in each condition. (b) Representative photomicrographs of phagocytosis by G*ϕ* developed after exposure to N, IH, or SH without (upper panel) or with (lower panel) NAC. (c) A representative analysis of intracellular latex beads localization using 3D reconstructing software IMARIS* z*-stack for 24 h IH-treated G*ϕ* in the presence of NAC. Upper panel in (c) represents an image of the G*ϕ*. Lower panel in (c) represents cross sections (*xy*, *xz*, and *yz*) for localization of the latex beads.

G*ϕ* were shown to internalize carboxylate-modified fluorescent yellow-green latex beads (1 *μ*m) more avidly than freshly isolated neutrophils. Yet, the IH-treated G*ϕ* (12 h, 29 cycles) exhibited a lower phagocytic activity (*p* = 0.015) compared to normoxic-G*ϕ* (Figure 9(a)), which was further attenuated in 24 h IH-treated G*ϕ* to about 50% of the ability of normoxic-G*ϕ* (*p* = 0.001) (Figures 9(a) and 9(b)). Treatment with NAC significantly increased this ability of normoxic-G*ϕ*, as well as that of the IH-treated G*ϕ* compared to the corresponding NAC-untreated G*ϕ*, whereas, in 24 h SH, NAC treatment had no effect on the phagocytosis compared to untreated G*ϕ* (Figures 9(a) and 9(b)). To exclude possible cytospinning-dependent false-positive latex internalization, we used as negative controls G*ϕ* that were kept on ice and cytospins were prepared immediately or 2 h after adding latex beads. Also, uptake of the beads was blocked by inhibiting actin (cytochalasin B, 10 *μ*M). Additionally, the intracellular localization of latex beads was also validated in G*ϕ* by 3D* z*-stack images as illustrated in Figure 9(c).

Enhanced phagocytosis in NAC-treated neutrophils was previously shown in a number of studies. While treatment* in vitro* of human neutrophils with NAC enhanced their phagocytic ability [46], also oral administration of NAC* in vivo* to healthy individuals increased this ability [47]. Similarly, the phagocytosis of neutrophils was augmented in NAC-treated patients in the intensive care unit [48]. Thus, treatment with NAC restored the phagocytic activity of the developed G*ϕ* under IH similarly to increasing their size, the expression of LC3B, and that of NADPH oxidase subunits gp91-*phox* and p22-*phox*. This may indicate that phagocytosis is one of the mechanisms contributing to the formation of G*ϕ*, as already indicated by restoring the formation of G*ϕ* under IH in the presence of NAC.

The importance of NAC in restoring, at least partially, G*ϕ* phenotype and functions in IH-treated cells may have clinical relevance to conditions associated with cyclic-intermittent hypoxia. NAC that is best known for treating glutathione deficiency [44] was also shown to exert protective effects in various systems including neuroprotection in ischemic stroke [49]. Treatment with NAC was also shown to affect the IH associated with OSA [16]. For instance, in animal models mimicking OSA treated with NAC, oxidative stress and liver inflammation were attenuated [50], pancreatic *β* cells were protected from apoptosis [51], pharyngeal muscle dilator performance was also improved [52], and diaphragm muscle dysfunction was prevented [53]. In addition, oral administration of NAC to patients with OSA improved sleep efficiency, shortened the duration of apneas, decreased lipid peroxidation, and increased total reduced glutathione [54]. Taken together, these findings demonstrate the protective effects exerted by the antioxidant NAC against IH-induced oxidative stress, inflammation, and overall improving OSA associated consequences.

## 4. Conclusions

Previously we have described for the first time a new subpopulation of giant phagocytes (G*ϕ*) derived from neutrophils maintained in prolonged culture conditions [25]. Exposing PMN to acute intermittent hypoxia (IH), common in inflammatory conditions, cancer, and sleep apnea, hampered G*ϕ* development in a dose-dependent manner whereas sustained hypoxia had no (or minimal) effect on their development. Intermittent hypoxia reduced G*ϕ* size, autophagy, neutrophil elastase and NADPH oxidase expression, and their phagocytic activity. Inhibiting NADPH oxidase or the PI3K/Akt signaling pathway completely abolished G*ϕ* development at all oxygen conditions investigated, indicating their importance for this process. Conversely, the antioxidant N-acetylcysteine (NAC) abrogated the IH-associated effects and partially restored a control like phenotype and functions.

The physiological/pathophysiological significance of G*ϕ* development* in vivo* is yet unknown. However, our earlier findings indicate their potential involvement in atherogenic processes by demonstrating their ability to internalize oxidized LDL (oxLDL) and to generate ROS in response to oxLDL uptake, unlike fresh neutrophils [25]. In accord with this line we have recently identified the presence of G*ϕ* in carotid plaques from patients undergoing elective endarterectomy. Thus, a better understanding of G*ϕ* formation may provide insights into basic neutrophil biology in inflammatory and atherogenic conditions or in the resolution of neutrophilic inflammation. Moreover, the a priori low yield of G*ϕ* may indicate that they have a unique function and may represent a subgroup of progenitor cells. However, their identification and roles* in vivo* warrant intensive investigation.

## Figures and Tables

**Table 1:** Effects of hypoxia on giant phagocytes (G*ϕ*) area and markers.

Measures	N	IH	SH
Area of cells (*µ*m^2^) (*n* = 9)	1486 ± 129	1051 ± 101^†,††^	1527 ± 158

Neutrophil elastase			
relative % (*n* = 3)^*∗*^	100 ± 36.4	16.7 ± 3.8^†,††^	58.1 ± 23.6
FI^#^	35,888 ± 13,326	4,847 ± 1,003	19,383 ± 7,304

LysoTracker			
relative % (*n* = 3)^*∗*^	100 ± 47.1	31.7 ± 9.1^†,††^	116.7 ± 51.7
FI^#^	17,501 ± 10,900	3,023 ± 1,748	21,633 ± 12,869

MitoTracker			
relative % (*n* = 3)^*∗*^	100 ± 40	59.7 ± 25.6^†,††^	98.2 ± 43.4
FI^#^	11,582 ± 4,183	6,284 ± 2,759	11,459 ± 6,063

LC3B			
relative % (*n* = 5)^*∗*^	100 ± 33.8	59.2 ± 20.8^†,††^	81.8 ± 27.7
FI^#^	34,246 ± 10,319	15,912 ± 6,962	27,078 ± 9,928

gp91-*phox *			
relative % (*n* = 5)^*∗*^	100 ± 46.2	28.4 ± 15.4^†,††^	65.1 ± 30.9
FI^#^	19,886 ± 8,975	5,506 ± 2,459	13,072 ± 6,844

p22-*phox *			
relative % (*n* = 3)^*∗*^	100 ± 41.5	45.0 ± 18.4^†,††^	165.3 ± 54.9^†††^
FI^#^	18,384 ± 9,569	5,772 ± 2,962	34,938 ± 11,756

Freshly isolated PMN were exposed for 24 h to intermittent hypoxia (IH), sustained hypoxia (SH), or normoxia (N). Then, cells were cultured at normoxia for additional six days. Area of cells and fluorescence intensity (FI), defined as Raw integrated density (sum of pixel values)/Area of cells, was integrated with Image J 1.49k Software as indicated in Materials and Methods.

^**∗**^FI at normoxia was considered as 100% and the effects of IH and SH were calculated as relative % of normoxia for each of the indicated number of experiments.

^#^FI representative data of one independent experiment. In each experiment at least 10 cells were counted at each condition.

^†^Significance IH versus N, *p* < 0.005.

^††^Significance IH versus SH, *p* < 0.005.

^†††^Significance SH versus N, *p* < 0.01.
